# Novel Angiotensin-Converting Enzyme-Inhibitory Peptides From Fermented Bovine Milk Started by *Lactobacillus helveticus* KLDS.31 and *Lactobacillus casei* KLDS.105: Purification, Identification, and Interaction Mechanisms

**DOI:** 10.3389/fmicb.2019.02643

**Published:** 2019-11-28

**Authors:** Jiaqi Li, Jiajia Zhao, Xindi Wang, Abdul Qayum, Muhammad Altaf Hussain, Guizhao Liang, Juncai Hou, Zhanmei Jiang, Aili Li

**Affiliations:** ^1^Key Laboratory of Dairy Science, Ministry of Education, Food Science College, Northeast Agricultural University, Harbin, China; ^2^Bioengineering College, Chongqing University, Chongqing, China

**Keywords:** angiotensin-converting enzyme inhibitory peptides, fermented bovine milk, isolation, sequence identification, molecular interaction

## Abstract

Fermented milks with strong angiotensin I-converting enzyme (ACE)-inhibitory activity were obtained through a culture with *Lactobacillus helveticus* KLDS.31 and *Lactobacillus casei* KLDS.105 with a fermentation and storage temperature of 37 °C. Ultrafiltration fractions with a molecular weight less than 3 kDa in fermented milk whey exhibited the strongest inhibitory activity. Correspondingly, a gastrointestinal digestion experiment showed retention of the bioactivity of these fractions with pepsin and trypsin treatment. Four ACE-inhibitory peptides from fermented milk were isolated, purified by two-step reverse chromatography, and sequenced. Furthermore, the interaction mechanisms between ACE and four isolated peptides were investigated by a molecular docking method and the Independent Gradient Model. Experimental determination of IC_50_ was done to verify theoretical results. The inhibitory peptide interacted with ACE as follows: Lys-Pro-Ala-Gly-Asp-Phe > Lys-Ala-Ala-Leu-Ser-Gly-Met > Lys-Lys-Ala-Ala-Met-Ala-Met > Leu-Asp-His-Val-Pro-Gly-Gly-Ala-Arg.

## Introduction

Hypertension is a major chronic disease that threatens human health. Blood pressure regulation in the human body occurs through a series of complex pathways, many of which involve angiotensin-converting enzyme (ACE; Bernstein et al., 2014). ACE is involved in the quinine-nitric oxide (QNOS) and renin-angiotensin (RAS; Rice et al., 2004) regulatory systems (Suarez and Anon, 2019). ACE plays a critical role in these pathways to maintain blood pressure at a balanced level (Rice et al., 2004; Sanli et al., 2018). The inhibitory activities of ACE are considered to be an effective way to treat hypertension (Krichen et al., 2018). Many synthetic ACE inhibitors, which are used to regulate blood pressure during clinical treatment, present some safety hazards for humans, such as potential induced coughing, renal impairment, and allergic reactions (Alhaj, 2017). In the past few years, ACE inhibitors from natural sources have been investigated as alternatives to synthetic drugs as they have potentially fewer side effects (Fitzgerald and Meisel, 2000; Jiang et al., 2010). Milk protein is an important source of food-derived polypeptides, including hydrolyzed polypeptides with antioxidant (Power et al., 2013), ACE-inhibitory (Hernández-Ledesma et al., 2010), antibacterial, and anti-cancer (Khan et al., 2018) bioactivity.

ACE-inhibitory peptides from bovine milk protein have been described previously (De Leo et al., 2009). These bioactive peptides are typically produced by enzymatic hydrolysis, microbial fermentation, or genetic engineering. In particular, more attention has been focused on microbial fermentation as the food grade bacteria, such as lactic acid bacteria (LAB), provide enriched food bioactive substances (Castellano et al., 2013). Furthermore, the hydrolytic processing of milk protein through the action of chymosin and *Lactobacillus casei* can also improve cheese flavor (Lemieux et al., 2010). Many lactobacillus strains release ACE-inhibitory peptides from fermented milk, such as Val-Pro-Pro-and Ile-Pro-Pro (Sipola et al., 2002; Mizushima et al., 2004; Hirota et al., 2007). Chen et al. (2007) fermented low-fat milk with several LAB (*Lb. casei, Lb. acidiphilis* and *Streptococcus thermophilus Lb bulgaricus*, *and Bifidobacterium*) and obtained two polypeptides (Gly-Val-Trp and Gly-Thr-Trp) with strong ACE-inhibitory activities (IC_50_ value of 240.0 and 464.4 μM). Pan and Guo (2010) demonstrated ACE-inhibitory activity in sour milk fermented with *Lb. helveticus* LB10, and the bioactive peptide Arg-Leu-Ser-Phe-Asp-Pro [from β-lactoglobulin hydrolysate (f148-153)] was isolated and purified, and it exhibited an IC_50_ value of 177 μM. In another report, ACE-inhibitory activity was found in a bioactive fraction (containing six peptides) from milk fermented with *Lactococcus lactis* DIBCA2 (IC_50_ = 5 ± 2 μg/mL) (Nejati et al., 2013). Overall, several studies have demonstrated the efficacy of microbial fermentation for production of ACE-inhibitory peptides.

In our previous study, two wild strains were obtained from traditional Chinese fermented dairy byproducts (such as sour soup) and identified by 16S rRNA gene sequencing analysis as *Lactobacillus helveticus* KLDS.31 and *Lactobacillus casei* KLDS.105. Fermented bovine milk prepared with *Lactobacillus helveticus* KLDS.31 and *Lactobacillus casei* KLDS.105 exhibited strong ACE-inhibitory and proteolytic activity. However, the amino acid sequence of these bioactive peptides and the mechanism through which these peptides interacted with ACE were not elucidated. In this work, ultrafiltration and two-step reverse phase chromatography are applied to isolate the peptides, and the amino acid sequences are then identified by mass spectrometry. The binding energy and hydrogen bonding between inhibitory peptides and ACE are calculated by molecular docking method, and the Independent Gradient Model (IGM; Lefebvre et al., 2017) is used to investigate the intermolecular mechanism. The results will reveal the mechanism by which these peptides interacted with ACE through the combination of theoretical and experimental methods, providing the basis for the further development and medical application of ACE-inhibitory peptides.

## Materials and Methods

### Chemical and Microorganisms

*Lactobacillus helveticus* KLDS.31 (No. 2805) and *Lactobacillus casei* KLDS.105 (No. 2806) were previously isolated and are stored at China General Microbiological Culture Collection Center (CGMCC), Institute of Microbiology, Chinese Academy of Sciences, Beijing, China. Hippuric acid, angiotensin I-converting enzyme (ACE), hippuryl-L-histidyl-L-Leucine (Hip-His-Leu), pepsin (EC.3.4.23.1, 1:10,000) and trypsin (Gibco-BRL, EC.3.4.21.4, 1:250, activity 2–4 U/mg) were obtained from Sigma Chemical Co., Ltd. (St. Louis, MO, United States). Ultrapure water was prepared by Milipore ultrapure Water System (Milipore Corporation, United States). High performance liquid chromatography grade trifluoroacetic acid and acetonitrile were from Merck & Co., Inc. (Germany).

### Preparation of Fermented Milk With ACE-Inhibitory Action

Freeze dried *Lactobacillus helveticus* KLDS.31 and *Lactobacillus casei* KLDS.105 were individually propagated in sterile lactobacilli Man-Rogosa-Sharpe (MRS) broth at 37°C for 24 h and then inoculated (1%, v/v) into 10 milliliter of sterile skim milk (protein 3.1%; lactose, 4.8%; fat, 0.2%). and incubated at 37°C for 24 h. The resulting cultures were used to inoculate (1%, v/v) 100 mL volumes of sterilized skim milk and were incubated at 37°C until milk curd formed. Three batches of fermented milk were produced under the sterilized condition. Fermented milk was centrifuged at 6,000 × *g* for 0.5 h and the obtained supernatant was used to determine ACE-inhibitory activity by high-performance liquid chromatography (HPLC, Waters, United States).

### ACE-Inhibitory Activity Determination

The determination was measured by HPLC, with slight changes according to the procedure described by Gonzalez-Gonzalez et al. (2011). The sample (20 μL) was dissolved in 120 μL Hip-His-Leu (5 mM) with sodium borate buffer (50 mM, pH 8.1). After incubation at 37°C for 3 min, the ACE solution (0.1 U/mL, 10 μL) was added to the sample and incubated for 60 min at the above temperature. Subsequently, 0.15 mL HCl (1 M) was used to terminate the reaction. The content of hippuric acid (HA) produced by reaction was determined by reverse-phase high-performance liquid chromatography (RP-HPLC, Waters Corporation, United States). A symmetry C18 Column (3.9 × 150 mm, 5 μm, Waters, United States) was used with the Waters HPLC model Alliance 2690 system, which included an ultraviolet detector (Waters, United States). A linear gradient from 10–60% acetonitrile in 0.1% trifluoroacetic acid (TFA) was applied in 10 min, then from 60% to 10% acetonitrile in 0.1%TFA was reached in 2 min. Flow rate was 0.8 mL/min. The sample (6 μL) was filtered (0.45 μm filter) and measured at 228 nm. A standard curve was generated using concentrations of standard HA and peak areas. The ACE-inhibitory activity was determined as:

$$\mathrm{ACE}\mathrm{inhibitory}\mathrm{activity}\left(\%\right)=\frac{{\left[H\,A\right]}_{c\,o\,n\,t\,r\,o\,l}-{\left[H\,A\right]}_{s\,a\,m\,p\,l\,e}}{{\left[H\,A\right]}_{c\,o\,n\,t\,r\,o\,l}}\times 100\%$$

The concentration of inhibitor that inhibits 50% ACE activity is defined as IC_50_ value.

### Effects of Fermentation and Storage Temperature on the ACE-Inhibitory Activity of Fermented Milk

An inoculum (1%, v/v) of *Lactobacillus helveticus* KLDS.31 and *Lactobacillus casei* KLDS.105 from the culture was transferred into sterilized skim milk and incubated at 37 and 42°C to ferment until curd formed. Additionally, fermented curds were refrigerated at 0–4°C or kept at 37°C for 24 h. The effects of the two fermentation temperatures and storage conditions on the inhibitory activity of the fermented milk were determined.

### Ultrafiltration Separation of ACE-Inhibitory Peptides

The sample was centrifuged at 6,000 × *g* for 0.5 h to collect the upper whey. The Pall-filtration system (Pall Corporation, United States) was used to separate the whey fractions of fermented milk. After aqueous whey was ultrafiltered with 10 kDa ultrafiltration membrane (Millipore Co., Billerica, MA, United States), ultrafiltration permeates (less than 10 kDa in size) and retentates (more than 10 kDa in size) were collected. The ultrafiltration permeates (less than 10 kDa) were further separated with a 3 kDa ultrafiltration membrane (Millipore Co., Billerica, MA, United States). Fractions of ultrafiltration permeates (between 3 and 10 kDa) and retentates (more than 3 kDa) were obtained. Different fractions (more than 10 kDa, less than10 kDa, 3 and 10 kDa, and less than 3 kDa) were collected to investigate ACE-inhibitory activity.

### Effect of Pepsin and Trypsin on Bioactivity of ACE-Inhibitory Peptides in Fermented Milk

As reported (Bhaskar et al., 2019), the obtained ultrafiltration permeates (less than 3 kDa) from fermented milk were used to hydrolyze together with pepsin and trypsin. Initially, ultrafiltration permeates (less than 3 kDa) from fermented milk were digested with 2% pepsin at pH 2.0 at 37°C for 1.5 h. After the hydrolysis of pepsin, this peptide mixture was heated at 95°C for 15 min. Subsequently, 2% trypsin was used to continuously hydrolyze the peptide mixture at pH 8 and 37°C for 4h. After trypsin hydrolysis, the enzymes were deactivated (95°C) for 15 min, and the ACE-inhibitory activities of the lyophilized hydrolysates were measured.

### Purification of ACE-Inhibitory Peptides in Fermented Milk

The obtained lyophilized ultrafiltration permeates (less than 3 kDa) from fermented milk were isolated with a Source 5RPC ST 4.6/150 column (Amersham Pharmacia) in a purification system (AKTA purified 100, Amersham Biosciences). Initially, 10 mM ammonium acetate buffer (pH 8.0) consisting of 2% acetonitrile as the mobile phase A was used to equilibrate this column. Next, a steeper gradient was carried out from 0 to 80% mobile phase B (70% acetonitrile) for 10 column volumes (CV). The flow rate and detection wavelength were set at 0.5 mL/min and 215 nm, respectively. The collected fractions were lyophilized, and its inhibitory activity was measured. Fractions with the strongest activity were further purified with the same reverse-phase column. The linear gradient was conducted from 0 to 30 % mobile phase B at the same flow rate and detection wavelength. Finally, collected fractions were lyophilized and their activities were measured. The step of bioactive sample collection was repeatedly performed.

### Sequence of the Purified Peptides Determination

The isolated ACE-inhibitory peptides from fermented milk exhibited high purity. The peptides were analyzed with the 4700 proteomics analyzer and matrix-assisted laser desorption and ionization-time of flight (MALDI-TOF/TOF) mass spectrometer (AB4700 proteomic analyzers). The samples (0.5 μL) were spotted onto target plates after mixing with MALDI matrix. Acetonitrile (0.5 μL; 0.5 g/L) with 0.1% of TFA was scattered after the samples were dried. An equipment of 200 Hz Nd/YAG laser was required and set at 355 nm. The parameters of the mode of reflectron-positive ion with delayed extraction were as follows: (MS mode) reflector positive parameter CID (OFF), mass range (700–3200 Da), focus mass (1200 Da), fixed laser intensity (6000), bin (10 ns), (MS/MS mode) IKV positive parameter, and CID(NO) precursors mass window near 80% resolution (FWMH) tight laser intensity. The 4700 Explorer software was used to control the instrument and the data were processed by GPS Explorer software and the Mascot platform.

### Molecular Docking and IGM-Based Visualize and Quantify Intermolecular Interaction

The structure of four polypeptides were optimized by a Gaussian 09 (Frisch et al., 2009) (B3LYP/6-31G) level of theory (Liu et al., 2017) until no imaginary frequency existed in the optimized structure. The receptor was download from the RCSB Protein Data Bank (PDB ID: 3BKK) (Watermeyer et al., 2008). The preparation of ACE mainly removed ligands and water from the crystal structure. The polar hydrogen of the ACE structure was added, and zinc ion and chloride ion were retained. The protonation states of the charged amino acid residues were set as default values. Docking was performed with a Surflex-dock program with Sybyl 8.1 software. The complex conformation was selected depended on the Total-Score and CSCORE (Consensus Scores, ≥3) (Tao et al., 2017). Methodological approaches of the molecular interaction of ACE and inhibitory peptides was performed by Multiwfn 3.7 (Lu and Chen, 2012) and VMD 1.9.3. The IGM method was performed a 3D isosurface and it visualized the intermolecular and intramolecular interaction by calculating the density gradient of each of the atoms. The weak molecular interaction was represented as the δg function, which can be divided into intermolecular interaction (δg^inter^) and intramolecular interaction (δg^intra^):

$$f\,\delta \,g=\delta \,g^{\text{inter}}+\delta \,g^{\text{intra}}$$

The isosurface of each complexes system was set at 0.004, and the upper and lower limit of color scale were set as −5.0 to 5.0.

### Statistics

Data of ACE-inhibitory activity are showed as mean and standard deviation. Analysis of the variance (ANVOA) was applied to assess the significant difference (*P* > 0.5) among the mean from triplicate values and was identified by applying a Duncan multiple test through the SSPS system software 22.

## Results and Discussion

### Effect of Fermentation and Storage Temperature on ACE-Inhibitory Activity

**FIGURE 1:**
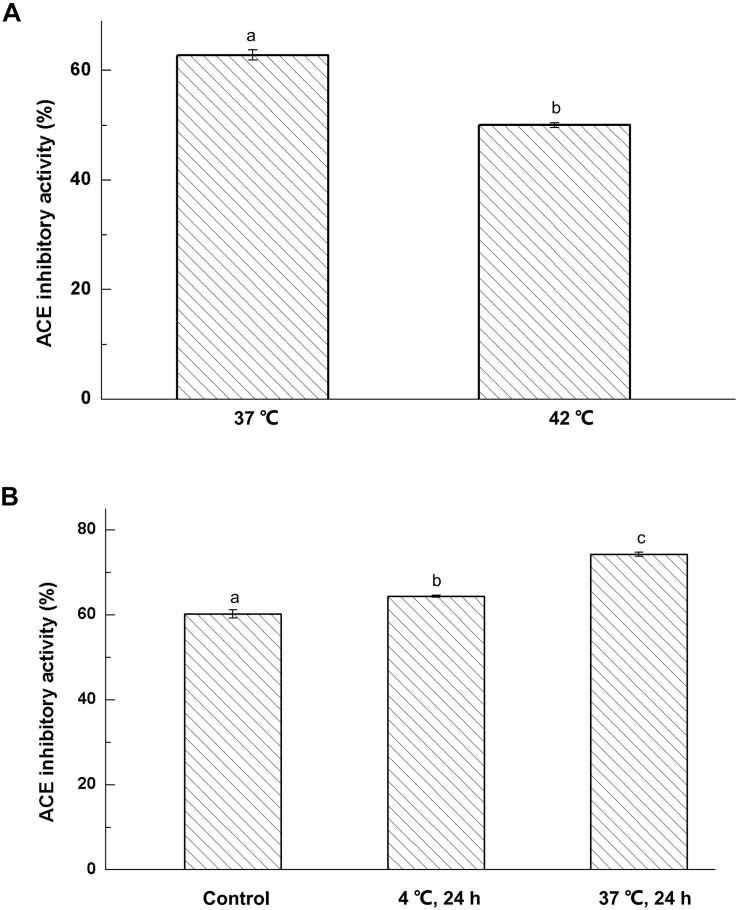
Effect of fermentation temperature **(A)** and storage conditions **(B)** on ACE-inhibitory activity of fermented milk; The column data with different letters means significant difference (α = 0.05).

Fermentation temperature has a great influence on the bioactivity of fermented milk (Li et al., 2015). In Figure 1A, the ACE-inhibitory activity in fermented milk at 37 °C was stronger than that obtained from cultures incubated at 42°C (*P* < 0.05). This meant that low temperature was more conductive to the hydrolysis of milk protein to produce bioactive peptides. The result also indicated ACE-inhibitory peptides can be cultured by *Lactobacillus helveticus* KLDS.31 and *Lactobacillus casei* KLDS.105 at 37°C.

Fermented milk was prepared from cultures of *Lactobacillus helveticus* KLDS.31 or *Lactobacillus casei* KLDS.105, and the cultures were then kept at 4 and 37°C for 3 days, respectively. The effect of storage temperature on ACE-inhibitory activity of fermented milk is shown in Figure 1B. The ACE-inhibitory activity of fermented milk was greatly enhanced at 4 and 37°C for 72 h (*P* < 0.05). Furthermore, the ACE-inhibitory activity of fermented milk kept at 37°C was remarkably higher than that at 4°C. This indicated that use of a high storage temperature would help to produce ACE-inhibitory peptides in fermented milk.

### Ultrafiltration Separation of ACE-Inhibitory Peptides in Fermented Milk

**FIGURE 2:**
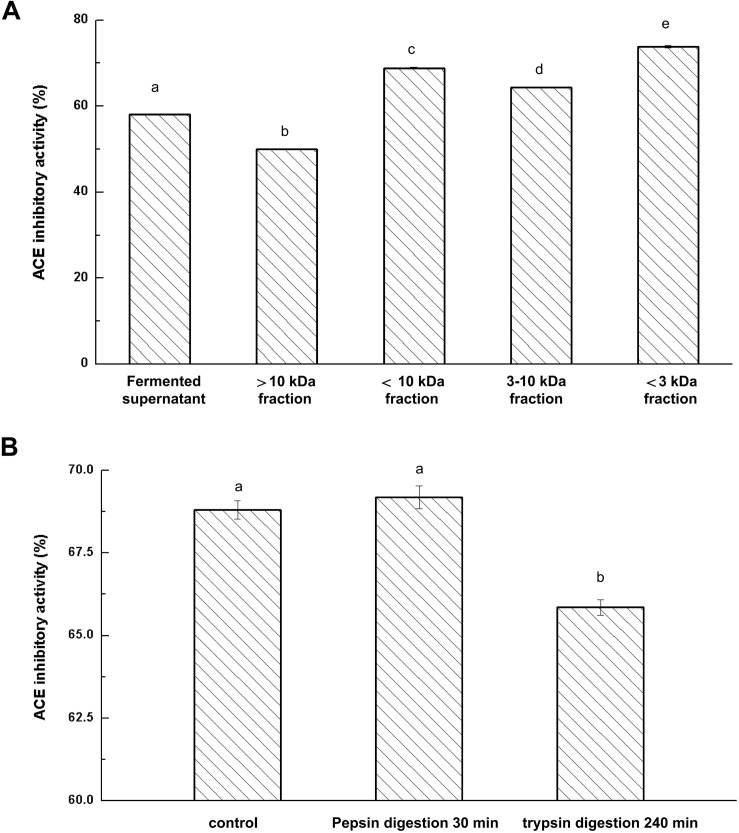
Effects of ultrafiltration **(A)** and Enzyme hydrolysis **(B)** on ACE-inhibitory activity of fermented milk; The column data with different letters means significant difference (α = 0.05).

ACE-inhibitory peptides produced in the fermented milk were separated with 10 and 3 kDa ultrafiltration membranes. Effects of ultrafiltration separation on the ACE-inhibitory activity of fermented milk were determined and the results are presented in Figure 2A.

After the ultrafiltration separation of the whey fraction in fermented milk, there were significant differences in the ACE-inhibitory activities among the fractions (*P* < 0.05). The fraction (less than 3 kDa) showed the strongest ACE-inhibitory activity and fraction (more than 10 kDa) represented the weakest ACE-inhibitory activity. These results indicated that ACE-inhibitory peptides with lower molecular weight had higher ACE-inhibitory activity. Rui et al. (2013) also reported that the fraction with low molecular weight peptides (less than 5 kDa) represented a significantly stronger activity than the retentate. Moreover, IC_50_ value of soybean protein hydrolysates increased three times after ultrafiltration (Wu and Ding, 2002). Thus, ultrafiltration played a significant role in the removal of peptides with large molecular weight and helped to enhance the ACE-inhibitory activity of isolates of fermented milk.

### Effect of Pepsin and Trypsin on Bioactivity of ACE-Inhibitory Peptides

Fermented milk was prepared by co-fermentation of the combined strains KLDS.31+KLDS.105 and the resulting culture was centrifuged to obtain the whey liquid. The 3 kDa fraction was separated through a 3 kDa ultrafiltration membrane. The ability to resist gastrointestinal digestion is of great importance because hydrolysis occurs throughout gastrointestinal digestion. Pepsin and trypsin were used to hydrolyze the obtained ultrafiltration permeates (less than 3 kDa in size) from fermented milk. The inhibitory activities of ACE-inhibitory peptides in milk after digestion by pepsin and trypsin are shown in Figure 2B. Pepsin treatment had no effect on ACE-inhibitory activity (*P* > 0.05). Additionally, the ACE-inhibitory activity only decreased by 3.91% after trypsin digestion, suggesting that the ACE-inhibitory peptides in the fermented milk retained original activity after digestion with pepsin and trypsin and thereby indicating that possible oral intake would retain the bioactivity of these hydrolysate components (Kazuhito et al., 2008).

### The ACE-Inhibitory Peptides Isolation

#### One-Step Reverse Phase Separation

**FIGURE 3:**
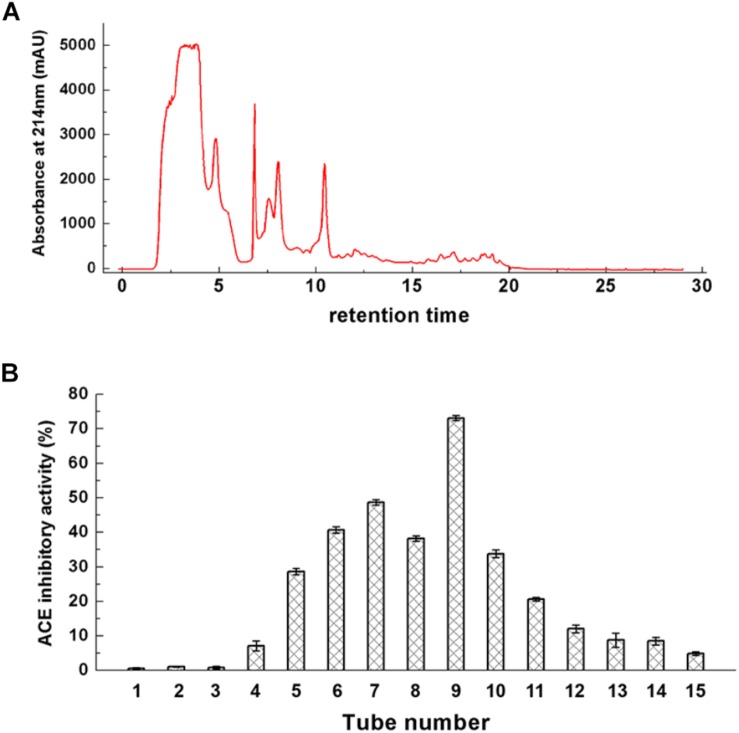
Chromatogram of ultrafiltrate with less than 3 kDa **(A)** and its ACE-inhibitory activity **(B)**.

Ultrafiltrate of a size less than 3 kDa was separated from fermented milk and was purified by reverse-phase chromatography. The chromatographic separation results are shown in Figure 3A. These ACE-inhibitory peptides were subjected to a gradient elution. The elute fractions exhibited wide and strong absorption peaks. Accordingly, five strong absorption peaks and several weaker absorption peak bands appeared. The eluted fraction was obtained and lyophilized, and their ACE-inhibitory activity in each elution tube was determined. Figure 3B showed the ACE-inhibitory activity of each elution tube. Six fractions showed stronger ACE-inhibitory activity (from No. 5 to No. 10). In particular, elution tube No. 9 exhibited powerful ACE-inhibitory activity and was recorded as a Z component. The Z component was repeatedly eluted, collected, and lyophilized.

#### Two-Step Reversed Phase Separation

**FIGURE 4:**
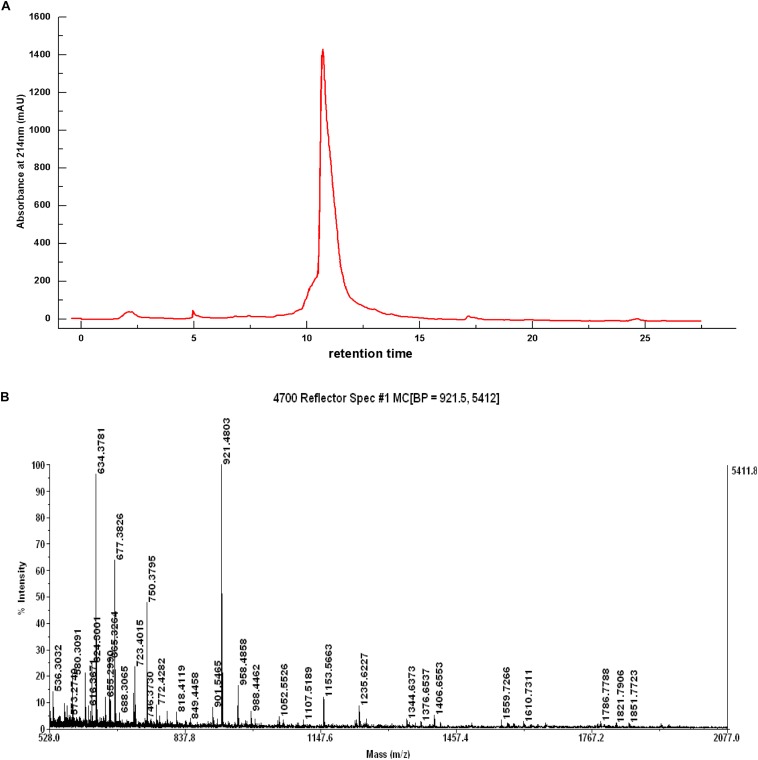
Chromatogram of Z component by the source TM 5RPC ST 4.6/150 column **(A)** and Mass Spectrum of the Z-I Composition **(B)**.

The Z component was continuously purified using the same reverse-phase column. A narrow elution gradient was selected for two-step purification. The chromatographic separation results of Z component are presented in Figure 4A. A strong absorption peak and several weak absorption peaks were shown in the chromatographic separation. Moreover, the IC_50_ value of the elution tube component with a strong absorption peak was 0.332 mg/mL, and it was recorded as Z-I component. After two-step purification, the obtained Z-I component had a high purity. This experiment fully confirmed that reversed-phase chromatography is an excellent technical method for the purification of polypeptide. Park et al. (2000) also used a variety of chromatographic techniques to isolate and purify the component with strong inhibitory activity from watercress.

### Amino Acid Sequence Analysis of ACE-Inhibitory Peptides

**FIGURE 5:**
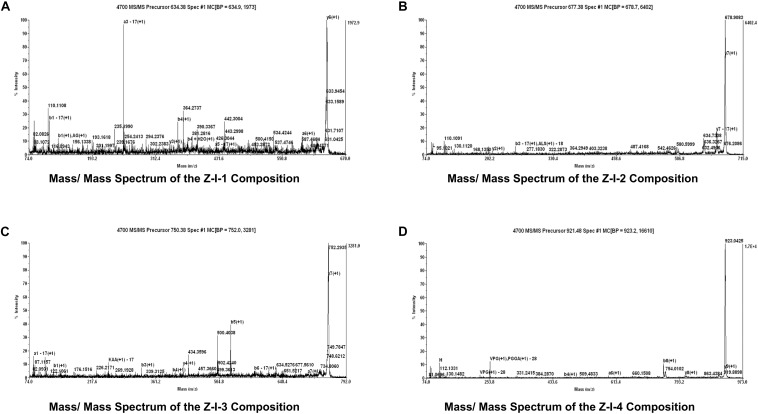
Mass/Mass Spectrum of the Z-I-1 **(A)**, Z-I-2 **(B)**, Z-I-3 **(C)**, and Z-I-4 **(D)**.

Mass spectrometry (Figure 4B) showed that the component Z-I has four main absorption peaks, 634.37, 677.38, 750.37, and 921.48 Da, which are recorded as Z-I-1, Z-I-2, Z-I-3, and Z-I-4. This indicated that the component Z-I was not a single component but instead contained four small peptides. Furthermore, our work showed the molecular mass values for four of the ACE-inhibitory peptides were less than 1 kDa. The MS/MS spectrometry analysis of the four components was shown in Figure 5. Additionally the sequence of these four ACE-inhibitory peptides are Lys-Pro-Ala-Gly-Asp-Phe (Z-I-1), Lys-Ala-Ala-Leu-Ser-Gly-Met (Z-I-2), Lys-Lys-Ala-Ala-Met-Ala-Met (Z-I-3), and Leu-Asp-His-Val-Pro-Gly-Gly-Ala-Arg (Z-I-4). Krichen et al. (2018) reported the extraction of four ACE-inhibitory peptides from shrimp and showed that hydrophobic amino acids at the N-terminus might contribute to an increase in the ACE-inhibitory activity of peptides. Here, Leu-Asp-His-Val-Pro-Gly-Gly-Ala-Arg similarly has leucine at the N-terminal end. Generally, the branched chain aliphatic amino acid and the aromatic amino acid at the N-terminal and C-terminal could act as a potential inhibitor of ACE (Cheung et al., 1980). For Lys-Pro-Ala-Gly-Asp-Phe, Lys-Lys-Ala-Ala-Met-Ala-Met, and Lys-Ala-Ala-Leu-Ser-Gly-Met, the presence of phenylalanine and methionine residues at the C-terminal end supported this point of view.

### Analysis of the Interaction of ACE With Isolated Polypeptides

**FIGURE 6:**
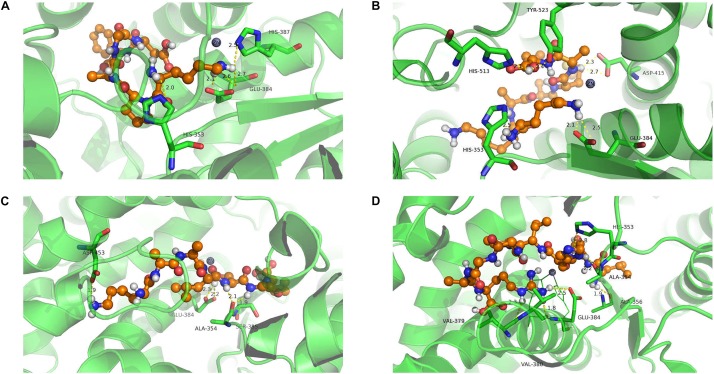
Molecule docking and hydrogen bond of ACE with isolated polypeptides (the hydrogen bond is represented by a yellow dotted line). Lys-Pro-Ala-Gly-Asp-Phe **(A)**; Lys-Lys-Ala-Ala-Met-Ala-Met **(B)**; Lys-Ala-Ala-Leu-Ser-Gly-Met **(C)**; Leu-Asp-His-Val-Pro-Gly-Gly-Ala-Arg **(D)**.

Molecular docking was used to investigate the binding affinity of inhibitory peptides with ACE. The binding structure with the highest Total-Score was generated and is presented in Figure 6. Each complex exhibits five hydrogen bonds at the binding site, of which zinc ion constitutes a tetrahedrally coordinated Zn (II) with ACE residues. From Figure 6, residues His353, Glu384, and His387 have a higher frequency of occurrence than other residues at the binding site. These residues also formed hydrogen bonds with four inhibitory polypeptides. Among them, Lys-Pro-Ala-Gly-Asp-Phe represented the largest Total-Score (8.49), having the strongest binding affinity compared with Lys-Ala-Ala-Leu-Ser-Gly-Met (7.35) and Lys-Lys-Ala-Ala-Met-Ala-Met (5.98). The binding affinity of the nonapeptide with ACE represented the weakest (Total-Score: 5.92). When inhibitory peptides interacted with the binding site, hydrogen bonds formed between ACE residues and inhibitory peptides, which influenced the tetrahedrally coordinated Zn (II) and represented the inhibitory activity of ACE (Tao et al., 2017). We considered the strong interaction of the hexapeptide because of its hydrogen bonds with His 387, which was also linked with Zn(II). This also suggested that Zn(II) is important for the molecular interaction in these system (Wu et al., 2019).

**FIGURE 7:**
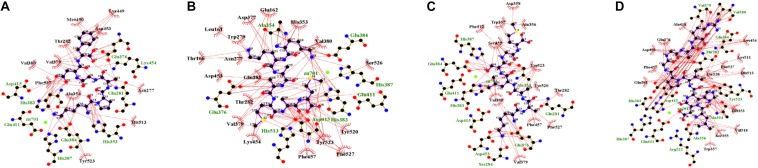
Hydrophobic interaction of binding site [Lys-Pro-Ala-Gly-Asp-Phe **(A)**; Lys-Lys-Ala-Ala-Met-Ala-Met **(B)**; Lys-Ala-Ala-Leu-Ser-Gly-Met **(C)**; Leu-Asp-His-Val-Pro-Gly-Gly-Ala-Arg **(D)**].

**FIGURE 8:**
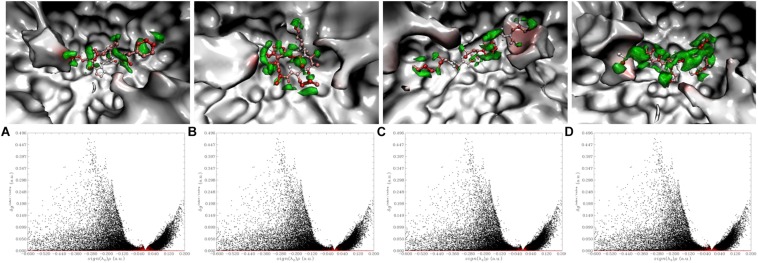
Visualize and quantify intermolecular interaction between inhibitory peptides with ACE by IGM [The upper figure: The more red the atom represents the larger δg indices; Green isosurface exhibited the interaction region. The lower figure: The red and black points correspond to δg^inter^ and δg^intra^, respectively. **(A)** Lys-Pro-Ala-Gly-Asp-Phe; **(B)** Lys-Lys-Ala-Ala-Met-Ala-Met; **(C)** Lys-Ala-Ala-Leu-Ser-Gly-Met; **(D)** Leu-Asp-His-Val-Pro-Gly-Gly-Ala-Arg].

In the enzyme-peptide interaction system, except the hydrogen bond described above, hydrophobic interaction, and van der Waals interaction also contribute to the enzyme–peptide interaction (Chen et al., 2019). Figure 7 visualizes the hydrophobic interaction in the binding site. Hydrophobic residues, Phe527, Tyr523, Glu411, His387, Glu384, His383, Glu376, Val379, Ala354, and Gln281 play a great role in all the systems. As shown by the scatter plot of δg with sign(λ_2_)ρ (Figure 8, the lower figure), the region around −0.04 on the X-axis exhibits a peak in four maps, suggesting hydrogen bonds exist in the complexes. Furthermore, the δg indices and van der Waals forces of the complex system are also presented (Figure 8, the upper figure). The green isosurfaces in these complex systems represent the hydrogen bonding and van der Waals forces in these systems. The color of atoms shows the contribution degree to intermolecular interaction. The atoms of peptides near to ACE residues showed more red, indicating the strong interaction between these atoms.

We also determined the IC_50_ of these four inhibitory peptides: Lys-Pro-Ala-Gly-Asp-Phe (77.45 ± 2.74 μM) > Lys-Ala-Ala-Leu-Ser-Gly-Met (110.35 ± 3.87 μM) > Lys-Lys-Ala-Ala-Met-Ala-Met (189 ± 4.61 μM) > Leu-Asp-His-Val-Pro-Gly-Gly-Ala-Arg (201 ± 5.24 μM), which represents the same order of Total-Score, and this verified the theoretical experiment.

## Conclusion

Fermentation of bovine milk using *Lactobacillus helveticus* KLDS.31 and *Lactobacillus casei* KLDS.105 yields a fermented milk that is rich in peptides with ACE-inhibitory activity. There were more ACE-inhibitory peptides produced at 37°C compared to cultures grown at 42°C. Moreover, compared with storage at 4°C, fermented milk had higher ACE activities when incubated at 37°C for 72 h (*P* < 0.05).

After ACE-inhibitory peptides derived from the fermented milk were digested with pepsin and trypsin, almost 94% of its initial activity remained. The purification of four peptides was completed by ultrafiltration and two steps of consecutive reverse-phase chromatography. The purified peptides were sequenced and the enzyme–peptide interaction (namely hydrogen bonding, hydrophobic interaction, and van der Waals forces) was visualized and investigated. The binding affinity was in the order of Lys-Pro-Ala-Gly-Asp-Phe, Lys-Ala-Ala-Leu-Ser-Gly-Met, Lys-Lys-Ala-Ala-Met-Ala-Met, and Leu-Asp-His-Val-Pro-Gly-Gly-Ala-Arg.

The interaction mechanism between the inhibitory peptides and ACE helped to clarify the strong activity of peptide. It appears that these peptides with strong ACE-inhibitory activity have a potential application as natural and safe food-derived medicines.

## Data Availability Statement

All datasets generated for this study are included in the article/supplementary material.

## Author Contributions

JL contributed to data acquisition, analysis, and manuscript drafting. JZ contributed to milk fermentation and MS spectra analysis. XW and AQ performed the determination of ACE inhibitory activity. MH contributed the data statistics. GL revised the part of molecular simulation. ZJ and JH conceived the study. AL revised the manuscript.

## Conflict of Interest

The authors declare that the research was conducted in the absence of any commercial or financial relationships that could be construed as a potential conflict of interest.
